# Temporomandibular disorder as an indicator of altered physiological stress reactivity: a highly standardized experimental protocol

**DOI:** 10.1186/s12903-026-08227-4

**Published:** 2026-04-02

**Authors:** Anke Hollinderbäumer, Monika Bjelopavlovic, Peer W. Kämmerer, Christina Erbe, Jochen Hardt, Katja Petrowski

**Affiliations:** 1 Department of Oral and Maxillofacial Surgery - Facial Plastic Surgery, University Medical Center of the Johannes Gutenberg-University Mainz, Augustusplatz 2, Mainz, 55131 Germany; 2 Department of Prosthetic Dentistry, University Medical Center of the Johannes Gutenberg- University Mainz, Augustusplatz 2, Mainz, 55131 Germany; 3 Department of Orthodontic Dentistry, University Medical Center of the Johannes Gutenberg- University Mainz, Augustusplatz 2, Mainz, 55131 Germany; 4 Department of Medical Psychology and Medical Sociology, University Medical Center of the Johannes Gutenberg-University Mainz, Duesbergweg 6, Mainz, 55131 Germany

**Keywords:** temporomandibular disorders, TMD, stress, heart rate variability, HRV, Trier Social Stress Test, psychological stress induction

## Abstract

**Background:**

Temporomandibular disorders (TMDs) are frequently associated with psychological stress. Their prevalence appears particularly high in populations exposed to substantial stress, such as students during examination periods. While subjective assessments have linked TMDs to psychological stress, physiological evidence remains limited. Heart rate variability (HRV) is an established biomarker of autonomic regulation and stress reactivity and may therefore be useful for characterizing stress responses in individuals with TMD symptoms.

**Objective:**

This study investigated stress reactivity in students with and without TMD symptoms using HRV as a physiological marker within a standardized stress protocol.

**Methods:**

A total of 138 healthy students (aged 23–36 years) were recruited. TMD symptoms were assessed using a standardized protocol based on bruxism guidelines, focusing on palpation-evoked pain and limitations in mandibular range of motion. Psychological stress and depressive symptoms were assessed using the Perceived Stress Scale (PSS) and the Beck Depression Inventory-II (BDI-II), respectively. Acute stress was induced using the Trier Social Stress Test (TSST). HRV was recorded at baseline, during the pre-stress phase, and during stress exposure. RR intervals were analyzed using Kubios software, with the root mean square of successive differences (RMSSD) as the primary HRV parameter. Statistical analyses used linear mixed-effects models adjusted for potential confounders, including age, sex, and baseline stress levels.

**Results:**

TMD symptoms, particularly palpation-evoked pain, were significantly associated with psychological stress measures (*p* < 0.005). Students with TMD symptoms also showed higher depressive symptom scores (*p* < 0.002). Due to participant dropout, statistical power for HRV analyses was limited; however, effect estimates suggested a trend toward prolonged autonomic dysregulation in students reporting TMD-related pain.

**Conclusion:**

The findings provide preliminary physiological evidence linking TMD symptoms to psychological stress and indicate altered autonomic stress responses in students with TMD pain. Early symptom recognition and the integration of stress management strategies may be beneficial components of TMD care. Future research should incorporate additional biomarkers (e.g., cortisol) and use larger longitudinal designs to improve clinical applicability.

## Introduction

A growing body of research suggests that stress and psychological distress are among the most important factors contributing to the onset and progression of temporomandibular disorders (TMDs) [1]. The global prevalence of TMD has been estimated at 29.5%, while prevalence estimates for the “muscle pain” subtype vary widely across studies (10% − 7%) [2]. As TMD represents a multifactorial clinical condition, a biopsychosocial model is required to understand its development [2]. Psychological factors commonly incorporated in this model such as anxiety, somatization, and psychological stress appear to play a substantial role in TMD [3]. In one study, 65.2% of patients with TMD and related symptoms reported chronic stress [4]. Consistently, stress levels were significantly higher in patients with TMD and bruxism than in individuals without these conditions [5]. Several reports further indicate that TMD prevalence increases as patient age decreases. Among students, prevalence estimates vary considerably, ranging from 44.3% [6] to 90.1%, compared with 75.7% among faculty [7]. Students also reported more symptoms and significantly more parafunctional activities than TMD comprises disorders of the temporomandibular joint and the surrounding musculature that may lead to pain and functional imfaculty members [8] pairment in the head, neck, and shoulder region [9]. Because diagnosis depends on multiple factors and the term “TMD” serves as an umbrella label for a broad and heterogeneous symptom spectrum, a universally accepted definition is lacking. One definition proposed by the German Society for Functional Diagnostics and Therapy (DGFDT) states: “TMD includes pain and/or dysfunction of the masticatory muscles and/or temporomandibular joints and/or dysfunction of occlusion” [1]. Clinical manifestations range from mild discomfort to severe pain and dysfunction of the jaw and associated musculature. Symptoms may also include tooth grinding or clenching, headaches, migraine, and tinnitus. The etiology is likewise heterogeneous; TMD is generally considered multifactorial. In addition to psychosocial factors, potential contributors include injury or trauma to the temporomandibular joint, inappropriate or excessive mechanical loading, infections or inflammation, as well as osteoarthritis or arthritis [4]. Whereas occlusal disorders and dental morphology were historically considered primary causes, a substantial body of evidence now emphasizes psychosocial factors, particularly stress, as key correlates [1].

Stress may promote excessive activation of the masticatory muscles [6], increasing load on the temporomandibular joint and surrounding tissues and potentially resulting in pain, muscle tension, and restricted mandibular movement. Moreover, stress has been proposed as a contributing factor to bruxism [10]. Graner et al. (2018) investigated associations between mental health, stress, and TMD prevalence in Brazilian dental students. They reported a higher prevalence of TMD among dental students (20%–48%) than in the general population (20% − 25%) and found that psychological factors were significantly associated with TMD. Specifically, the risk of TMD was up to four times higher among students who had received psychiatric treatment before or during their studies and among those who rated their academic performance and social life more negatively. In addition, higher resilience was associated with a lower prevalence of TMD, suggesting a potential protective role [11].

Although prior studies indicate a clear association between TMD and stress [1, 4, 7, 12], objective physiological evidence - particularly based on heart rate variability (HRV) measured during standardized stress induction—remains limited.

HRV is a widely used marker of autonomic regulation and can provide information about an individual’s physiological stress state, reflecting the balance between parasympathetic and sympathetic activity and aspects of sympathetic–adrenal–medullary (SAM) axis activation [13]. HRV describes the variation in time intervals between consecutive heartbeats (RR intervals), which is regulated by the autonomic nervous system [14]. Higher HRV is generally interpreted as indicating healthier autonomic flexibility, whereas lower HRV may reflect reduced parasympathetic activity and autonomic imbalance and has been associated with stress, mental disorders, and cardiovascular disease [14, 15]. HRV is typically quantified from electrocardiographic recordings by analyzing RR intervals with high temporal precision [14, 15].

Empirical studies comparing patients with TMD to control groups have reported altered HRV in TMD, suggesting an association between TMD and autonomic regulation [16–18]. Some findings also indicate that TMD-focused therapeutic interventions may be accompanied by improvements in autonomic function, reflected in HRV changes. Conversely, interventions targeting autonomic regulation may exert beneficial effects on TMD-related symptoms. However, the underlying mechanisms and the directionality of these associations remain to be clarified.

It is still unclear whether and to what extent stress-related HRV reactivity is altered in individuals with TMD. Addressing this question requires HRV measurement under standardized conditions during stress induction, alongside a detailed assessment of TMD symptomatology. Therefore, the aim of the present study was to investigate whether stress reactivity, assessed using HRV during a standardized stress test, differs in individuals with TMD compared with those without TMD symptoms.

## Materials and methods

### Study participants

This pilot study included 138 healthy students recruited at Johannes Gutenberg University Mainz via electronic advertisements and snowball sampling [19]. After completion of the clinical and psychological assessments, 66 participants remained for the subsequent procedures. 60% of the participants were female. The age range was 23–36 years. Exclusion criteria were acute or chronic medical conditions, current or past psychiatric diagnoses, regular medication use, substance use, and major stressful life events within the previous six months. Heavy smoking (> 10 cigarettes/day) and age outside the predefined range (22–37 years) also led to exclusion. Eligibility was verified during a telephone screening using the Structured Clinical Interview for DSM Disorders (SCID) [20] based on the Diagnostic and Statistical Manual of Mental Disorders (DSM-IV) [21].

### Ethics

All participants received written study information and provided written informed consent. Participation was voluntary, and non-participation was not associated with any disadvantages. Participants were informed that they could withdraw at any time without providing reasons and without negative consequences. The participant information and consent materials, as well as the study protocol, were approved by the local ethics committee. Ethical approval was granted by the Ethics Committee of the Landesärztekammer Rheinland-Pfalz, Germany (No. 2019–14188).

### Materials

#### Clinical measures – TMD diagnosis

Clinical functional status was assessed using a structured questionnaire and standardized clinical criteria to determine the presence and impact of temporomandibular disorders (TMD). TMD assessment was standardized according to the German Society for Functional Diagnostics and Therapy (DGFDT) [1]. Participants were asked about and/or examined for:

- pain or functional impairment in head and neck regions,
- tenderness or pain on palpation of specific muscle groups,
- temporomandibular joint noises, and.
- pain or impairment during mandibular movements.

#### Psychological measures

Psychological status was assessed using seven instruments:

1. Structured Clinical Interview for DSM Disorders (SCID) [20]: a semi-structured diagnostic interview based on DSM (Diagnostic and Statistical Manual of Mental Disorders) criteria [21] used to assess mental disorders reliably and validly.
2. Symptom Checklist-90-Revised (SCL-90-R) [22]: a self-report instrument assessing psychological and somatic distress (90 items; five-point response format).
3. Perceived Stress Scale (PSS), German version [23]: 14 items rated on a five-point Likert scale (1 = never to 5 = very often).
4. Beck Depression Inventory-II (BDI-II) [24]: 21 items assessing depressive symptoms (0–3 per item; total score 0–63).
5. State–Trait Anxiety Inventory (STAI) [25]: assesses state and trait anxiety using two 20-item subscales rated on a four-point Likert scale (1 = not at all to 4 = very much).
6. Primary Appraisal Secondary Appraisal (PASA) [26]: assesses cognitive appraisal processes; primary appraisal reflects perceived threat; secondary appraisal reflects perceived coping resources. A stress index is computed as an integrated measure of transactional stress; higher scores indicate higher perceived stress. The PASA comprises 16 items rated on a six-point Likert scale (1 = completely disagree to 6 = completely agree).
7. Visual analogue scale (VAS): used to assess self-reported stress after each condition, ranging from 0 (no stress) to 100 (maximum stress).

#### Heart rate measurement

Continuous heart rate was recorded using the Polar V800 system with an H10 chest strap (Polar, Finland; sampling rate 1000 Hz). RR intervals were analyzed using Kubios software (Kubios Oy, Kuopio, Finland). The automated detection algorithm was used to identify and correct artifacts and ectopic beats (artifact correction threshold: 0.45 s). HRV was evaluated in 5-minute segments during the experimental and recovery phases: condition 1 (0–5 min), condition 2 (5–10 min), condition 3 (10–15 min), recovery 1 (0–5 min post-condition), and recovery 2 (5–10 min post-condition).

#### Trier social stress test

The Trier Social Stress Test (TSST) [27], according to Kirschbaum et al. (1993), is a standardized laboratory paradigm that reliably induces acute psychosocial stress. It combines two core stressors: social-evaluative threat, whereby participants’ performance is judged by others, and uncontrollability, elicited through unexpected tasks in an explicitly evaluative setting [27].

#### Procedures

First, the administrative formalities were completed, and the participants were given standardized instructions regarding the study protocol. This was followed by a waiting period of around 15 min. Participants were instructed to refrain from eating, drinking (except water, if permitted), and smoking for at least 2 h before testing and throughout the approximately 2-hour testing session. Overall, approximately 20 min were scheduled prior to TSST onset, including arrival and physiological set-up. During the waiting phase, participants completed the Symptom Checklist-90-Revised (SCL-90-R) [22] to confirm eligibility. Each participant was fitted with a chest strap for continuous heart rate recording, and the recording device was prepared. Subsequently, a 3-minute respiratory sinus arrhythmia (RSA) assessment was conducted, during which breathing was standardized to six paced breaths per minute. The steady rhythmic technique was used.

Physiological set-up and baseline (Room 1): Baseline measurements (T0) were obtained at the beginning of the waiting phase. After 15 min, the participant was escorted to the test room (Room 2), where the TSST panel was already present.

TSST phase (Room 2): Preparation: Tasks were explained using a standardized script. Participants received 5 min to prepare their speech (Condition 1). Free speech: The experimenter visibly initiated video recording and then left the room; the panel remained seated. Participants delivered a 5-minute speech, with standardized prompts provided by a panel member if necessary (Condition 2).

Mental arithmetic: Immediately after the speech, the panel introduced a 5-minute mental arithmetic task without prior warning. This unexpected transition was intended to enhance perceived uncontrollability (Condition 3).

Cognitive appraisal was assessed three minutes after the start of each condition using the Primary Appraisal Secondary Appraisal (PASA) questionnaire [26]. Immediately after each condition, self-reported stress was assessed using a visual analogue scale (VAS).

Return, debriefing, and recovery (Room 1): After completion of the arithmetic task, participants returned to Room 1 (Recovery 1), which occurred approximately 20 min after TSST onset. A VAS assessment was administered, followed by a standardized debriefing. Participants were informed that the tasks were designed to be difficult, that the camera and panel were essential components of the stress paradigm, and that no actual performance evaluation was intended (Recovery 2). Participants then remained in the waiting area for an additional recovery period of approximately 35 min; neutral magazines were provided.

**Fig. 1:**
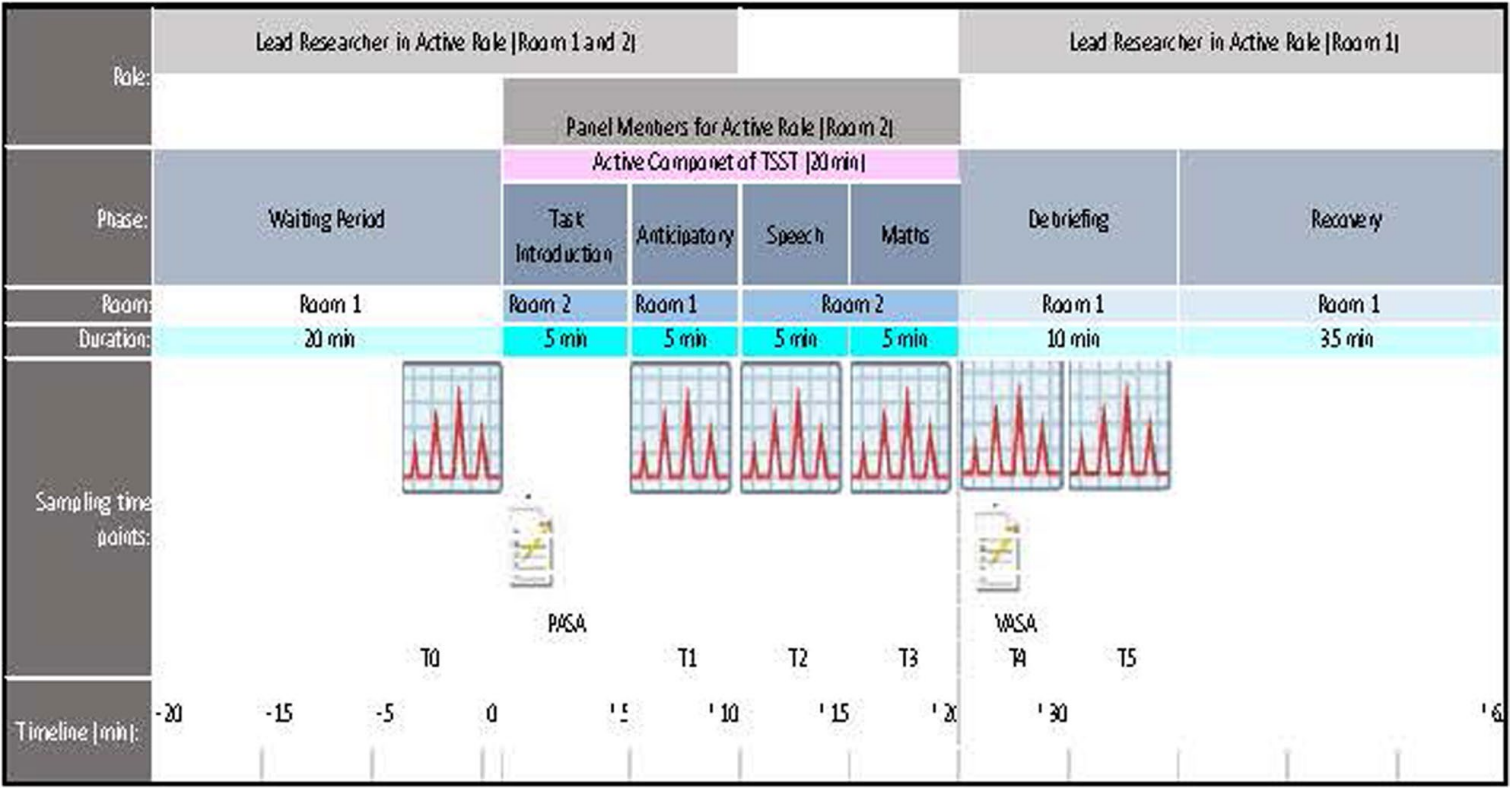
Overview of the examination procedure. PASA, Primary Appraisal Secondary Appraisal; TSST, Trier Social Stress Test; VAS, visual analogue scale. Based on Labuschagne et al. [28].

An overview of the examination procedure is presented in Fig. 1.

### Statistics

#### Statistical analysis

Descriptive statistics are reported as frequencies and percentages. Associations were examined using Spearman’s rank correlation coefficients. Between-group differences were assessed using two-tailed independent-samples t tests. The effect of acute stress on heart rate variability (HRV) was analyzed using a general linear model (GLM).

#### Software

Statistical analyses were performed using jamovi (The jamovi project, 2025; version 2.3.28) and IBM SPSS Statistics for Windows (IBM Corp., 2022; version 29.5).

## Results

### Evaluation of clinical functional status (TMD)

Between 12 and 16 students reported facial pain, whereas 36 to 41 reported no pain. Thirteen to fourteen participants did not provide information on this item.

Regarding temporomandibular joint palpation, 47 to 53 students provided data; of these, 3 to 8 reported discomfort and 1 to 2 reported pains, 13 to 19 students did not participate in this assessment.

### TMD criteria and the psychological symptoms

To represent the correlations between TMD criteria and psychological symptoms in Table 1, the TMD criteria were assessed in accordance with the DGFDT protocol.

**Table 1:**
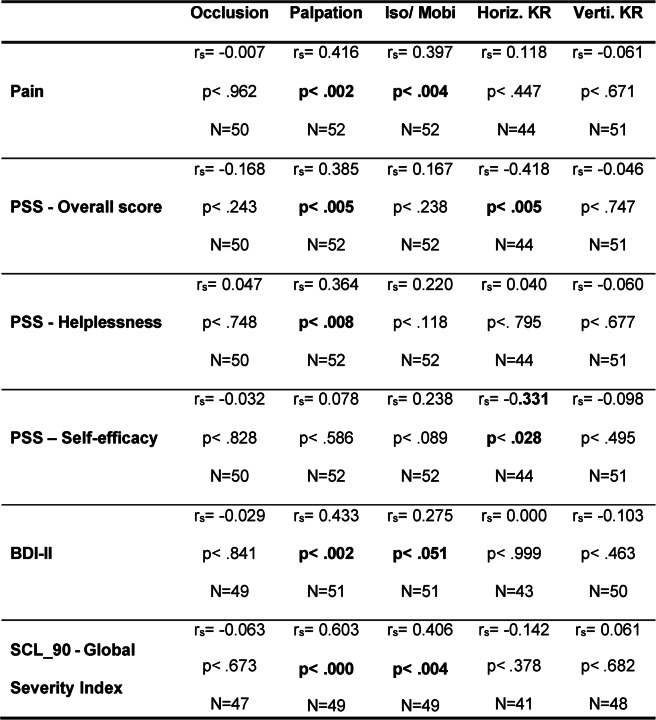
Correlations between TMD criteria and psychological symptoms

so/Mobi = Isometry/Mobility, Horiz.KR = Horizontal Jaw Relation, Verti.KR = Vertical Jaw Relation

Temporomandibular joint palpation was significantly correlated with psychological stress parameters, with the exception of negative self-efficacy. Significant correlations were also observed for isometry/mobility and horizontal jaw relation. No significant correlations with psychological stress parameters were found for vertical jaw relation.

### psychological stress induction

Following the TSST, participants completed the VAS immediately after the procedure and before the resting condition. Overall, 68% indicated that the statement “I found the situation challenging” was somewhat to completely true. Similarly, 75% rated the statement “The situation was stressful for me” as somewhat to fully accurate.

Perceived stress at the beginning of the TSST was significantly correlated with higher scores for temporomandibular joint palpation.

### Group comparison: students with vs. without TMD pain on perceived stress symptoms

Table 2 compares students who reported TMD pain with those who did not report TMD pain. The pain-related items listed in Table 3 were combined into a composite sum score (“pain”), which was used to classify participants into pain vs. no pain groups. These groups were compared with respect to psychometric measures of anxiety, stress, and depression.

**Table 2:**
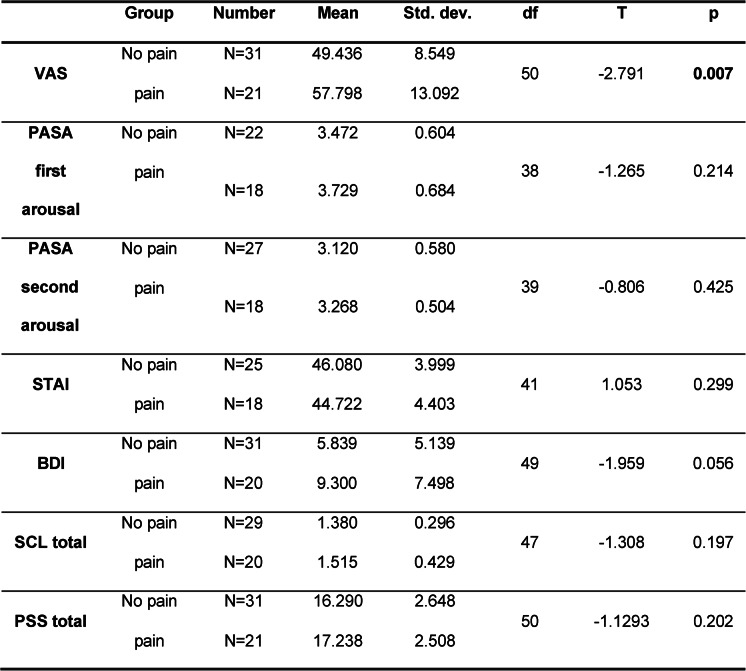
Students with TMD pain vs. students without TMD pain

**Table 3:**
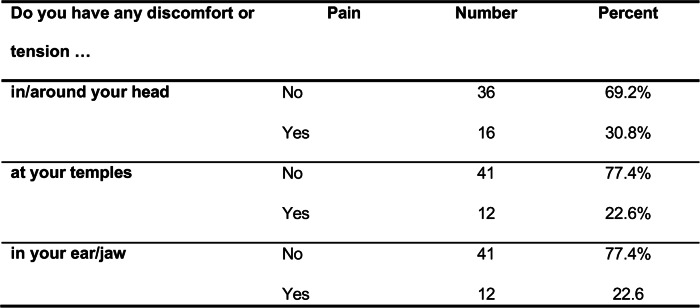
Facial pain reported in the functional diagnostic

assessment based on the DGFDT protocol

Table 2 shows significant group differences only for VAS Pain/No Pain for the BDI, a statistical trend is present (*p* = 0.056).

### Heart Rate Variability during stress induction

At TSST onset (T0), both groups showed increased RMSSD values. During the TSST, RMSSD decreased in both groups. Up to T2, RMSSD values in the TMD group were slightly lower than those in the control group. From T2 onwards, RMSSD values in the control group were lower than those in the TMD group. From T2 to T3, RMSSD values in both groups appeared to plateau.

## Discussion

The present study yielded several noteworthy findings. First, TMD-related clinical parameters, particularly temporomandibular joint palpation, were significantly associated with psychological stress symptoms, whereas vertical jaw relation showed no such relationship. Significant associations were also observed for isometry/mobility and horizontal jaw relation, suggesting that specific functional aspects of TMD may be more closely linked to psychological burden than others. Second, the TSST was perceived as stressful by the majority of participants, supporting the validity of the experimental stress induction. In this context, higher perceived stress at the beginning of the TSST was significantly correlated with increased temporomandibular joint palpation scores. Third, when students with and without TMD pain were compared, significant group differences were found only for the pain-related VAS measure, while depressive symptoms showed a statistical trend. Finally, HRV, indexed by RMSSD, decreased during the TSST in both groups, indicating autonomic stress reactivity. Although both groups showed a broadly similar decline over time, the pattern of RMSSD values differed slightly between students with and without TMD pain across the measurement points.

**Fig. 2:**
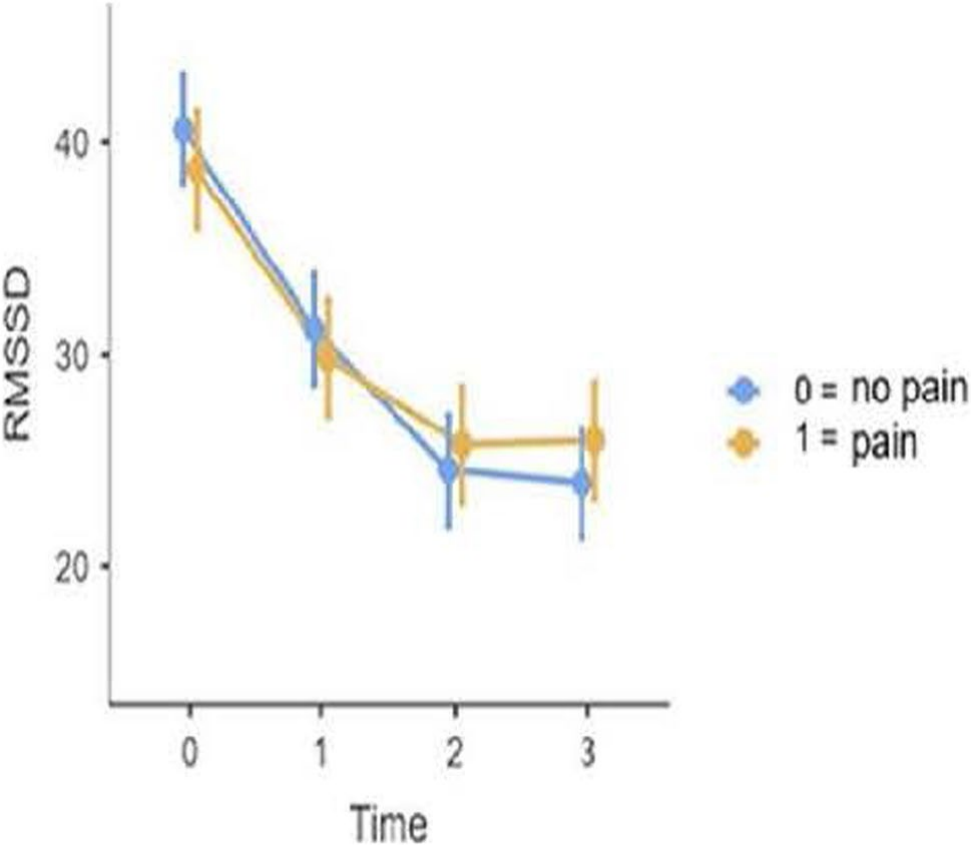
Change in stress levels in healthy students and students with TMD pain (TMD present), with 95% confidence intervals, plotted in relation to RMSSD

The relatively high prevalence of self-reported head and facial pain following administration of the TMD questionnaire (23–31%) suggests that a substantial proportion of the students may be affected by TMD symptoms [9]. The significant associations between stress-related symptoms and individual TMD criteria support the presence of a stress-related symptom profile. In the present study, the stress-related profile refers to the multidimensional pattern of cognitive, emotional, behavioral, and physical manifestations associated with stress exposure. It thus captures how stress is subjectively experienced and expressed at the individual level. Correlations were observed most frequently for palpation-based criteria (Table 4). Predominant abnormalities detected by palpation may indicate a rather mild or early manifestation of TMD, in which functional limitations or structural findings are not yet prominent. Administration of the TSST confirmed successful subjective stress induction (Table 1). Notably, significant associations between TMD criteria and stress parameters were already evident at the beginning of the TSST, suggesting an early coupling between TMD features and stress reactivity. In contrast, no significant between-group differences were found in heart rate variability. This is plausible given the reduced analyzable sample size and the resulting limited statistical power, particularly for HRV outcomes. The group-specific RMSSD trajectories shown in Fig. 2 should therefore be interpreted cautiously, as participant numbers fluctuated substantially across measurement time points and technical issues occurred, including missing recovery measurements (T4 and T5). Consequently, it remains unclear whether potential group differences would primarily relate to the acute stress response, recovery-phase regulation, or both. (Table 5).

**Table 4:**
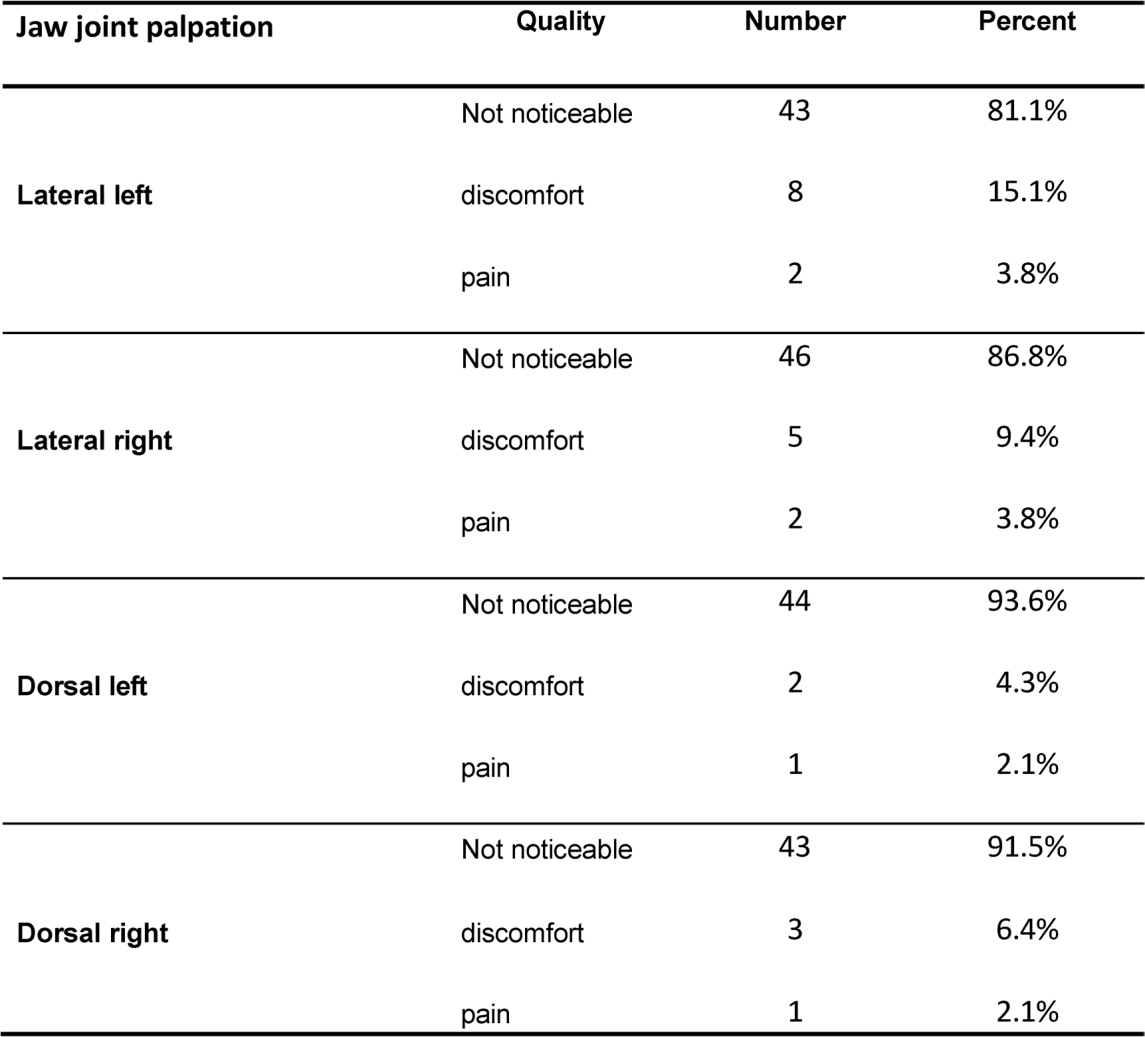
Temporomandibular joint palpation findings reported in the functional diagnostic

assessment based on the DGFDT protocol

**Table 5:**
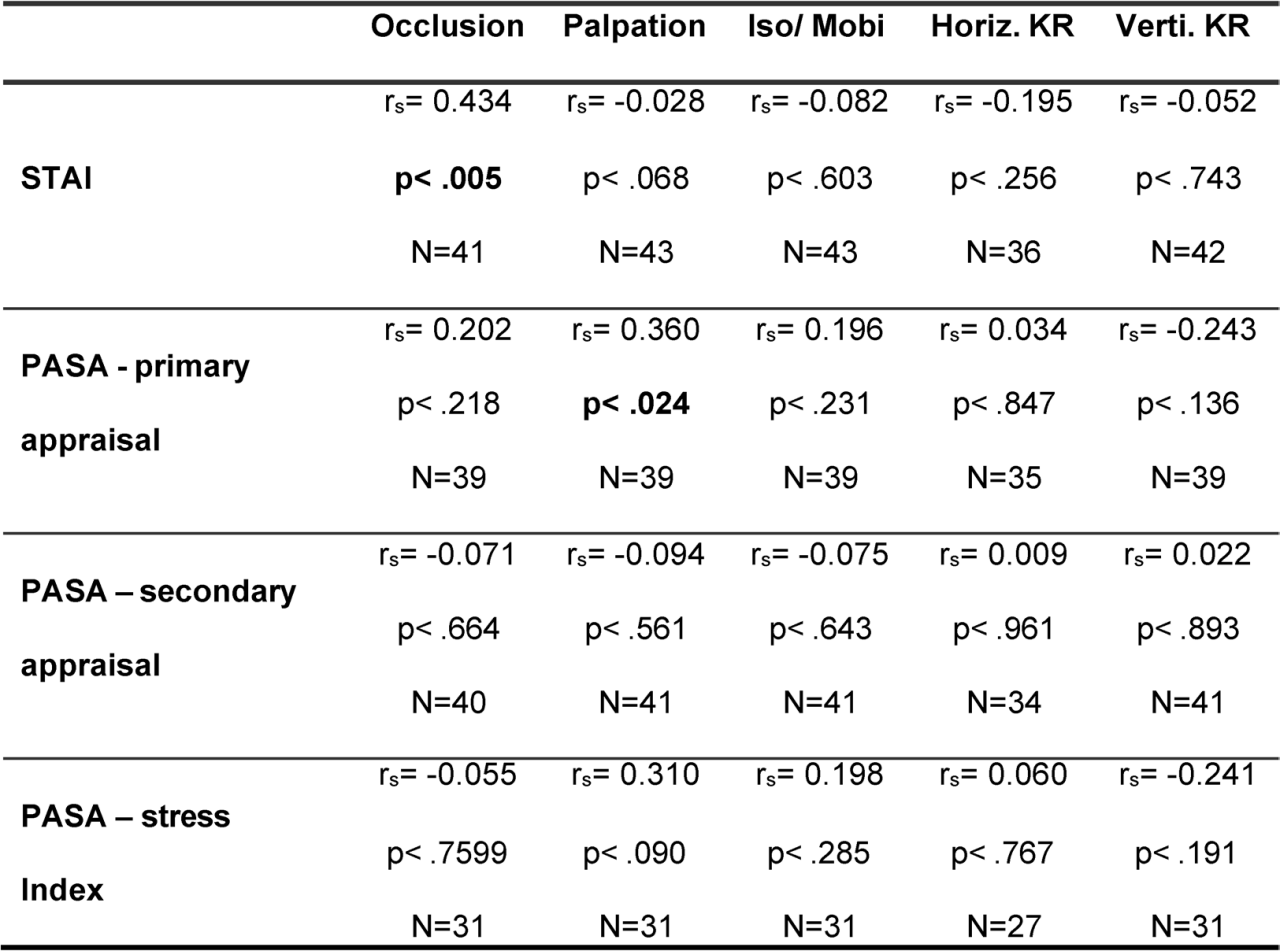
Spearman’s correlations between TSST-induced stress and TMD criteria

### Strengths

of this study include a relatively homogeneous cohort with a narrow age range, a socially comparable sex distribution, and standardized TMD assessment according to the DGFDT criteria [1]. Data collection by a single examiner likely reduced inter-examiner variability. In addition, the TSST, an established psychosocial stress paradigm, was implemented in this population to examine TMD-related stress responses under standardized conditions. (Table 6).

**Table 6:**
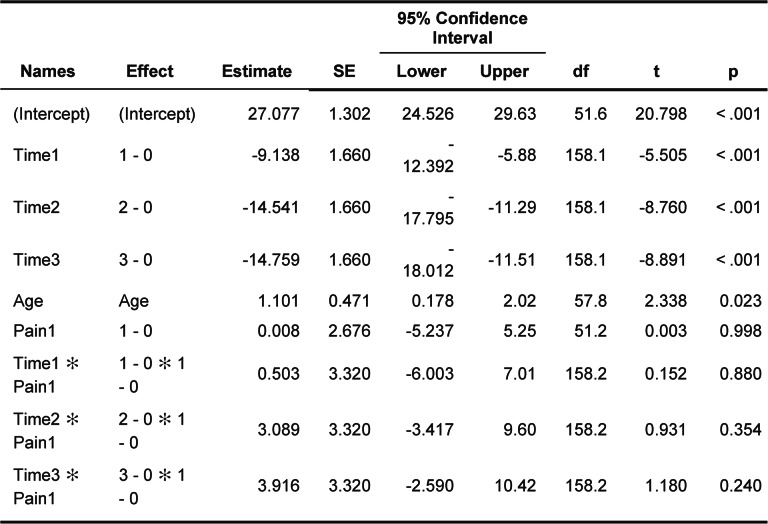
Fixed Effects parameter estimates

### Limitations

primarily involve the high dropout rate and the resulting lack of statistical power for several analyses, as well as deviations from the standardized TSST protocol during the recovery phase. For future studies, reducing participant burden by streamlining the psychometric assessment battery and focusing TMD assessment on core criteria may improve data completeness. With a larger fully analyzable sample, TMD subgroups (e.g., myofascial pain vs. predominant temporomandibular joint involvement) could be defined to examine differential stress-reactivity patterns and to refine the clinical interpretation of the findings.

## Conclusion

For future research, it would be desirable to distinguish between methodological implications and clinical relevance:

### Methodological implications

In addition to HRV, cortisol should also be assessed and analyzed as a further physiological marker of stress [29–31]. In laboratory studies, cortisol levels typically increase particularly when stressors involve social-evaluative threat and (perceived) uncontrollability [32]. The psychometric test battery should be reconsidered and reduced in order to minimize participant burden. These modifications may increase the likelihood of complete data collection and improve the analyzable sample size. With a larger sample, meaningful TMD subgroups (e.g., myofascial pain vs. temporomandibular joint disorders) could be examined to determine whether stress reactivity differs between clinical phenotypes.

### Clinical relevance

Since stress is likely to increase during examination periods and mild TMD symptoms may progress in susceptible students, preventive and supportive interventions should be considered, such as targeted stress management strategies (e.g., HRV biofeedback, progressive muscle relaxation, breathing therapy, mindfulness-based approaches, and yoga). Previous literature has described associations between stress and myogenous TMD [33–35]. Investigating these associations under standardized conditions, as enabled by the TSST, would strengthen the physiological interpretation. In addition, a longitudinal design could capture temporal changes in TMD symptoms in relation to stress reactivity.

Overall, the aim should be to identify mild cases at an early stage and to prevent progression to chronic conditions through the implementation of stress-reducing strategies.

## Data Availability

Data can be requested from the corresponding author.

## Abbreviations

TMD: Temporomandibular disorder
HRV: Heart rate variability
PSS: Perceived Stress Scale
BDI-II: Beck Depression Inventory-II
TSST: Trier Social Stress Test
RR: RR Interval ECG
RMSD: Root Mean Square of Successive Differences
DSM-IV: Diagnostic and Statistical Manual of Mental Disorders
SCID: Structured Clinical Interview DSM Disorders
DGFDT: German Society for Functional Diagnostics and Therapy
SCL-90-R: Symptom Checklist-90-Revised
STAI: State–Trait Anxiety Inventory
PASA: Primary Appraisal Secondary Appraisal
VAS: Visual analogue scale
GLM: General linear model

## References

[1] DGFDT. S3 Leitlinie Bruxismus. Available from: AWMF Leitlinien-Register S3-Leitlinie Diagnostik und Behandlung des Bruxismus. 02.05.2019. last access 2019;18(02):2025.

[2] Alqutaibi AY, Alhammadi MS, Hamadallah HH et al. Ammar Abdulrahman, Altarjami, Omar Talal Malloush, Aseel Mohammed Aloufi,. Global prevalence of temporomandibular disorders: a systematic review and meta-analysis. Journal of Oral & Facial Pain and Headache. 2025;39(2):48–65. 10.22514/jofph.2025.025. 10.22514/jofph.2025.025 PMC12531580 41070533

[3] Li Y, Zhang L, Cao Z, Sun Y, Xu Z, Marpaung C, et al. Targeted online health information was associated with more severe temporomandibular disorders. J Oral Facial Pain Headache. 2025;39(4):242–51. 10.22514/jofph.2025.081. 41436121 10.22514/jofph.2025.081 PMC12727180

[4] Pesqueira AA, Zuim PR, Monteiro DR, Ribeiro Pdo P, Garcia AR. Relationship between psychological factors and symptoms of TMD in university undergraduate students. Acta Odontol Latinoam. 2010;23(3):182–7. 21638957

[5] Fritzen VM, Colonetti T, Cruz MVB, Ferraz SD, Ceretta L, Tuon L, et al. Levels of Salivary Cortisol in Adults and Children with Bruxism Diagnosis: A Systematic Review and Meta-Analysis. J Evid Based Dent Pract. 2022;22(1):101634. 35219468 10.1016/j.jebdp.2021.101634

[6] Wolowski A, Schneider HJ, Eger T. Zahnmedizinische Beschwerdebilder mit psychosozialem Hintergrund [Dental disorders with a psychosocial background]. Bundesgesundheitsblatt Gesundheitsforschung Gesundheitsschutz. 2021;64(8):951–8. 10.1007/s00103-021-03369-y. German. doi:. 34212207 10.1007/s00103-021-03369-y PMC8316243

[7] Larkin N, Fricton V, Sangalli L, Prodoehl J, Fricton J. Prevalence and impact of signs and symptoms of temporomandibular disorders in dental students and faculty. J Dent Educ. 2024;88(12):1696–708. 10.1002/jdd.13675. Epub 2024 Jul 31. PMID: 39086000. 39086000 10.1002/jdd.13675

[8] Braas R, Eger T, Gohr J, Wörner F, Wolowski A. Orofaziale Funktionsstörung und posttraumatisches Belastungssyndrom: Eine Zusammenhangsanalyse bei Soldaten nach militärischen Einsätzen [Orofacial dysfunction and posttraumatic stress disorder : A context analysis of soldiers after military deployment]. Nervenarzt. 2019;90(5):503–508. German. 10.1007/s00115-018-0570-9. PMID: 30043219. 10.1007/s00115-018-0570-9 30043219

[9] Schmidt H, Hölzle F, Jackowski J. Craniomandibuläre Dysfunktionen. Zahnärztliche Chirurgie. 2017:711-38. 10.1007/978-3-642-54754-6_18.

[10] Katayoun E, Sima F, Naser V, Anahita D. Study of the relationship of psychosocial disorders to bruxism in adolescents. J Indian Soc Pedod Prev Dent. 2008;26(Suppl 3):S91–7. 19127024

[11] Graner KM, de Moraes ABA, Torres AR, Lima MCP, Rolim GS, Ramos-Cerqueira ATA. Prevalence and correlates of common mental disorders among dental students in Brazil. PLoS ONE. 2018;13(9):e0204558. 30261025 10.1371/journal.pone.0204558 PMC6160106

[12] Vlăduțu D, Popescu SM, Mercuț R, Ionescu M, Scrieciu M, Glodeanu AD, et al. Associations between Bruxism, Stress, and Manifestations of Temporomandibular Disorder in Young Students. Int J Environ Res Public Health. 2022;19(9):5415. 10.3390/ijerph19095415. 10.3390/ijerph19095415 PMC9102407 35564810

[13] Sztajzel J. Heart rate variability: a noninvasive electrocardiographic method to measure the autonomic nervous system. Swiss Med Wkly. 2004;134(35–36):514 – 22. 10.4414/smw.2004.10321. PMID: 15517504. 10.4414/smw.2004.10321 15517504

[14] Cygankiewicz I, Zareba W. Heart rate variability. Handb Clin Neurol. 2013;117:379–93. 24095141 10.1016/B978-0-444-53491-0.00031-6

[15] Sammito S, Thielmann B, Klussmann A, Deußen A, Braumann K-M, Böckelmann I. Nutzung der Herzschlagfrequenz und der Herzfrequenzvariabilität in der Arbeitsmedizin und der Arbeitswissenschaft. S2k-Leitlinie. AWMF-Register Nr. 002-042. Aktualisierte Fassung 2021/2022.

[16] Chinthakanan S, Laosuwan K, Boonyawong P, Kumfu S, Chattipakorn N, Chattipakorn SC. Reduced heart rate variability and increased saliva cortisol in patients with TMD. Arch Oral Biol. 2018;90:125–9. 29604544 10.1016/j.archoralbio.2018.03.011

[17] Santana MD, de Souza AC, de Abreu LC, Valenti VE. Association between oral variables and heart rate variability. Int Arch Med. 2013;6(1):49. 24373329 10.1186/1755-7682-6-49 PMC3879647

[18] Maixner W, Greenspan JD, Dubner R, Bair E, Mulkey F, Miller V, et al. Potential autonomic risk factors for chronic TMD: descriptive data and empirically identified domains from the OPPERA case-control study. J Pain. 2011;12(11 Suppl):T75–91. 22074754 10.1016/j.jpain.2011.09.002 PMC3233841

[19] Gabler S. Schneeballverfahren und verwandte Stichprobendesigns. ZUMA Nachr. 1992;16(31):47–69. https://nbn-resolving.org/urn:nbn:de:0168-ssoar. -210848 last access 18.02.2025.

[20] Wittchen HU, Wunderlich U, Gruschwitz S, Zaudig M. SKID-I. Strukturiertes Klinisches Interview für DSM-IV. Achse I: Psychische Störungen. Göttingen: Hogrefe; 1997.

[21] American Psychiatric Association. Diagnostic and statistical manual of mental disorders: DSM-IV. 4th ed. Washington, DC: American Psychiatric Association; 1994.

[22] Klaghofer R, Brähler E. Die Symptom-Checkliste von Derogatis (SCL-90-R). Deutsche Version von G. H. Franke. Göttingen: Beltz Test GmbH. 1995.

[23] Schneider EE, Schönfelder S, Domke-Wolf M, Wessa M. Measuring stress in clinical and nonclinical subjects using a German adaptation of the Perceived Stress Scale. Int J Clin Health Psychol. 2020;20(2):173–81. 32550857 10.1016/j.ijchp.2020.03.004 PMC7296237

[24] Herzberg P, Goldschmidt S, Heinrichs N. Beck Depressions-Inventar (BDI-II). Revision. Rep Psychologie. 2008;33(6):301–2.

[25] Laux L, Glanzmann P, Schaffner P, Spielberger C. D. Das State-Trait-Angstinventar (STAI): Theoretische Grundlagen und Handanweisung. Beltz Test. 1981.

[26] Gaab J. PASA–Primary Appraisal Secondary Appraisal-Ein Fragebogen zur Erfassung von situations-bezogenen kognitiven Bewertungen. Verhaltenstherapie. 2009;19(2):114–5.

[27] Kirschbaum C, Pirke K-M, Hellhammer DH. The ‘Trier Social Stress Test’–a tool for investigating psychobiological stress responses in a laboratory setting. Neuropsychobiology. 1993;28(1–2):76–81. 8255414 10.1159/000119004

[28] Labuschagne I, Grace C, Rendell P, Terrett G, Heinrichs M. An introductory guide to conducting the Trier Social Stress Test. Neurosci Biobehav Rev. 2019;107:686–95. 10.1016/j.neubiorev.2019.09.032. Epub 2019 Sep 24. PMID: 31560923. 31560923 10.1016/j.neubiorev.2019.09.032

[29] Klepzig K, Wendt J, Teusch L, Rickert C, Kordaß B, Lotze M. Pain and salivary biomarkers of stress in temperomandibular disorders were affected by maxillary splints. J Oral Rehabil. 2024;51(6):1025–33. 10.1111/joor.13678. Epub 2024 Mar 12. PMID: 38475974. 38475974 10.1111/joor.13678

[30] Papandreou M, Philippou A, Taso O, Koutsilieris M, Kaperda A. The effect of treatment regimens on salivary cortisol levels in patients with chronic musculoskeletal disorders. J Bodyw Mov Ther. 2020;24(1):100–8. 10.1016/j.jbmt.2019.10.010. Epub 2019 Oct 15. PMID: 31987528. 31987528 10.1016/j.jbmt.2019.10.010

[31] Brunyé TT, Goring SA, Navarro E, Hart-Pomerantz H, Grekin S, McKinlay AM, Plessow F. Identifying the most effective acute stress induction methods for producing SAM- and HPA-related physiological responses: a meta-analysis. Anxiety Stress Coping. 2025;9:1–23. Epub ahead of print. 10.1080/10615806.2025.2450620. PMID: 39788724. 10.1080/10615806.2025.2450620 39788724

[32] Dickerson SS, Kemeny ME. Acute stressors and cortisol responses: a theoretical integration and synthesis of laboratory research. Psychol Bull. 2004;130(3):355 – 91. 10.1037/0033-2909.130.3.355. PMID: 15122924. 10.1037/0033-2909.130.3.355 15122924

[33] Dammann J, Klepzig K, Schenkenberger E, Kordass B, Lotze M. Association of decrease in insula fMRI activation with changes in trait anxiety in patients with craniomandibular disorder (CMD). Behav Brain Res. 2020;379:112327. 10.1016/j.bbr.2019.112327. Epub 2019 Nov 4. PMID: 31697982. 31697982 10.1016/j.bbr.2019.112327

[34] Kuć J, Szarejko KD, Gołȩbiewska M, Smiling. Yawning, Jaw Functional Limitations and Oral Behaviors With Respect to General Health Status in Patients With Temporomandibular Disorder-Myofascial Pain With Referral. Front Neurol. 2021;12:646293. PMID: 34108927; PMCID: PMC8182059. 34108927 10.3389/fneur.2021.646293 PMC8182059

[35] de Leeuw JR, Steenks MH, Ros WJ, Bosman F, Winnubst JA, Scholte AM. Psychosocial aspects of craniomandibular dysfunction. An assessment of clinical and community findings. J Oral Rehabil. 1994;21(2):127 – 43. 10.1111/j.1365-2842.1994.tb01132.x. PMID: 8182495. 10.1111/j.1365-2842.1994.tb01132.x 8182495

